# The history of seabird colonies and the North Water ecosystem: Contributions from palaeoecological and archaeological evidence

**DOI:** 10.1007/s13280-018-1031-1

**Published:** 2018-03-07

**Authors:** Thomas A. Davidson, Sebastian Wetterich, Kasper L. Johansen, Bjarne Grønnow, Torben Windirsch, Erik Jeppesen, Jari Syväranta, Jesper Olsen, Ivan González-Bergonzoni, Astrid Strunk, Nicolaj K. Larsen, Hanno Meyer, Jens Søndergaard, Rune Dietz, Igor Eulears, Anders Mosbech

**Affiliations:** 1 Department of Bioscience, Arctic Research Centre, Aarhus University, Vejlsøvej 25, 8600 Silkeborg, Denmark; 2 Alfred Wegener Institute, Telegrafenberg A43, 14473 Potsdam, Germany; 3 Department of Bioscience, Arctic Research Centre, Aarhus University, Frederiksborgvej 399, 4000 Roskilde, Denmark; 4 The National Museum of Denmark, Frederiksholms Kanal 12, 1220 Copenhagen K, Denmark; 5 Department of Environmental and Biological Sciences, University of Eastern Finland, PL 111, 80101 Joensuu, Finland; 6 Department of Physics and Astronomy, Aarhus University, Ny Munkegade 120, Building 1522, 8000 Aarhus, Denmark; 7 Laboratorio de Etología, Ecología y Evolución, Instituto de Investigaciones Biológicas Clemente Estable, Av Italia 3318, 11600 Montevideo, Uruguay; 8 Institut for Geoscience, Aarhus University, Høegh-Guldbergs Gade, 2 bygning 1672, 115, 8000 Aarhus C, Denmark; 9 Department of Bioscience, Aarhus University, Frederiksborgvej 399, 4000 Roskilde, Denmark

**Keywords:** *δ*^15^N, Greenland, Little auk, Palaeoecology, Palaeolmnology

## Abstract

The North Water (NOW) polynya is one of the most productive marine areas of the Arctic and an important breeding area for millions of seabirds. There is, however, little information on the dynamics of the polynya or the bird populations over the long term. Here, we used sediment archives from a lake and peat deposits along the Greenland coast of the NOW polynya to track long-term patterns in the dynamics of the seabird populations. Radiocarbon dates show that the thick-billed murre (*Uria lomvia*) and the common eider (*Somateria mollissima*) have been present for at least 5500 cal. years. The first recorded arrival of the little auk (*Alle alle*) was around 4400 cal. years bp at Annikitsoq, with arrival at Qeqertaq (Salve Ø) colony dated to 3600 cal. years bp. Concentrations of cadmium and phosphorus (both abundant in little auk guano) in the lake and peat cores suggest that there was a period of large variation in bird numbers between 2500 and 1500 cal. years bp. The little auk arrival times show a strong accord with past periods of colder climate and with some aspects of human settlement in the area.

**Electronic supplementary material:**

The online version of this article (10.1007/s13280-018-1031-1) contains supplementary material, which is available to authorized users.

## Introduction

**Fig. 1:**
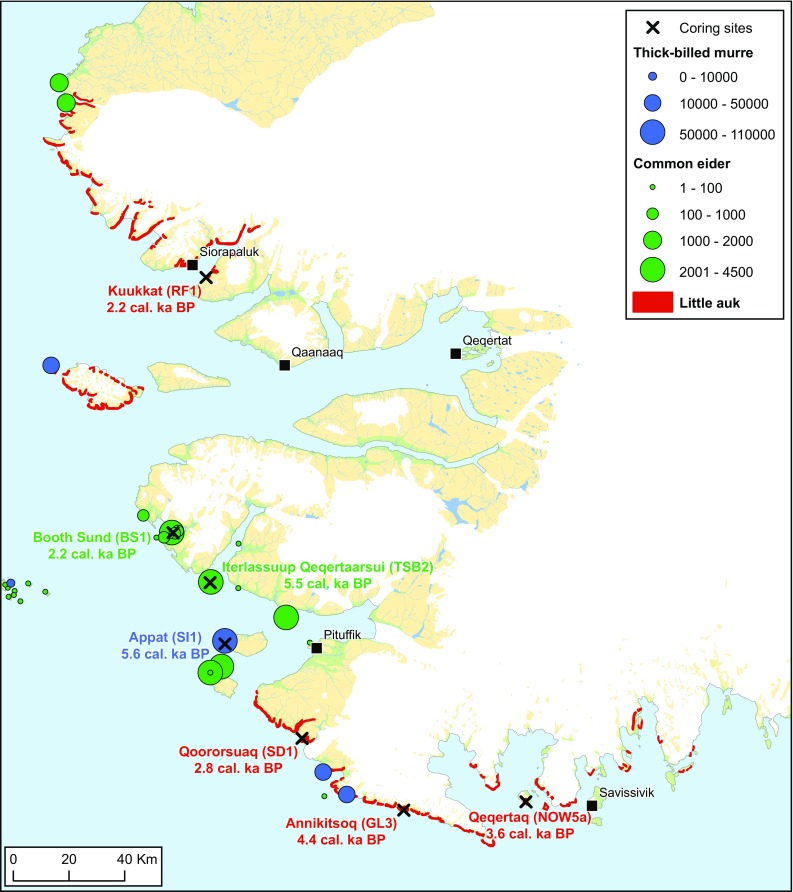
Overview map of the coring sites and breeding colonies of little auk, Qoororsuaq (Søkongedalen—SD1), Annikitsoq, Great Lake—GL-3 and Kuukkat (Robertson fjord—RF1), Qeqertaq (Salve Ø—NOW5a) thick-billed murre (Saunders Ø—SI-1) and common eider Booth Sund—BS-1 and Iterlassuup Qeqertaarsui (Three Sister Bess—TSB-2). For the latter two species, colony sizes are given as number of breeding pairs. Colony data come from Boertmann and Mosbech (1998) and The Greenland Seabird Colony Register, maintained by Danish Center for Environment and Energy, Aarhus University, and Greenland Institute of Natural Resources. The estimated date of arrival is the median cal. ka years bp calculated from ^14^C dates and age modelling (see “Materials and methods”)

The North Water polynya (NOW) marine ecosystem is host to the largest seabird populations in Greenland. The community is diverse with 14 regular breeders and a few more species occurring as non-breeding summer visitors. The seabirds are almost exclusively present in the spring and summer, with the exception of some black guillemots (*Cepphus grylle*), which can winter in the NOW. Here, we focus on the three most abundant seabird species: the little auk (*Alle alle*), the thick-billed murre (*Uria lomvia*), and the common eider (*Somateria mollissima*) (Fig. 1). These species have the largest biomass and the greatest importance of the locally harvested seabird species. The breeding population of the little auk in the NOW region is immense, estimated at 33 million pairs (Boertmann and Mosbech 1998; Egevang et al. 2003) and corresponding to more than 80% of the global breeding population. The thick-billed murre colonies along the Greenland coast of the NOW consist of approximately 225 000 breeding pairs (Merkel et al. 2014) representing two thirds of the breeding population in Greenland. Currently, the NOW is the only area in Greenland where the thick-billed murre population is not in decline. The common eider breeding population in the NOW was estimated at 25–30 000 pairs in 2009, and has had a fivefold increase between 1997 and 2009 (Burnham et al. 2012). This increase is related to the stricter harvest regulations which came into force in 2001, especially the restricting of spring harvest near the colonies, which sparked a general population increase in all West Greenland populations following a decline in the 20th century related to overharvesting (Merkel 2010). Thus, despite the large uncertainties in the estimates, the NOW is clearly of great international importance to the species.

Information on the past seabird populations in the High Arctic is generally scarce, with a few investigations providing some information on past populations. A study from the east coast of Greenland (Wagner and Melles 2001) used lake sediments to track the history of a nearby little auk colony over much of the Holocene. They found that large numbers of birds arrived ca. 7500 cal. year bp, but suggest that they have not been continuously present since. The colony appears to have been present there from 7500 to 1900 cal. bp, from 1000 to 500 cal. bp, and then again over the last 100 years to the present. The authors suggest that the absence of birds was related to periods of colder climate in East Greenland. Expanding to the wider Arctic, there is evidence of the presence of Arctic tern (*Sterna paradisaea*), glaucous gull (*Larus hyperboreus*), and barnacle goose (*Branta leucopsis*) in Svalbard as far back as 9400 cal. bp (Yuan et al. 2009). Palaeoecological studies in the Canadian Arctic found no evidence of variation in the northern fulmar (*Fulmarus glacialis)* colonies over the short time period (maximum 200 years) covered by the sediment cores (Michelutti et al. 2009; Keatley et al. 2011). Work on peat deposits in Hudson Bay showed that the thick-billed murre colonies were at least 1500 and 3800 years old (Gaston and Donaldson 1995). In the NOW area, there is very little information on the past populations of seabirds. A study using the sedimentary archive of peat deposits on the Carey Islands demonstrated the presence of a seabird colony, probably the Atlantic puffin (*Fratercula arctica*), during the period from c. 7100 to 5100 cal. bp (Bennike et al. 2007). Apart from this study, there is no information available on the arrival times and change in abundance of seabirds in the NOW within a long-term perspective.

Seabirds feed in the marine ecosystem, but use the terrestrial environment for breeding. As a result, some species transport large quantities of nutrients from sea to land, which transforms the landscape around the colonies, leaving unequivocal signatures of their presence (González-Bergonzoni et al. 2017; Mosbech et al. 2018). These signatures in the landscape, both terrestrial and fresh water, open up the possibility of investigating past dynamics of seabird populations. The reliance of our three species upon the marine ecosystem varies and largely depends on their different feeding strategies and habitats.

All three species require open water in which to feed upon arrival in spring. Thus, all three species benefit from the early season open water of the polynya. The little auk is a zooplanktivore and is highly dependent on the abundant copepods within their diving range (approx. 50 m) and foraging range from the colonies (approx. 100 km). They feed these large, lipid-rich copepods to their chicks (Frandsen et al. 2014) and also rely on the copepods for their own foraging, supplemented with larger zooplankton (Karnovsky et al. 2008). The high density of large *Calanus* copepods is a key factor driving the abundance of the little auk colonies in the NOW area. The diet of the adult thick-billed murre during summer is dominated by the pelagic amphipod (*Parathemisto libellula*) and arctic cod (*Boreogadus saida*), supplemented with a variety of other invertebrates and fish (Gaston and Hipfner 2000; Karnovsky et al. 2008). It performs pursuit dives down to 150 m of depth within a foraging range of approx. 110 km around the breeding colonies (Mosbech et al., unpublished GPS-tracking data). Even though adult murres feed on a variety of food items, they are strongly dependent on abundance of forage fish for breeding success. The murre is a “single-prey loader”, capable of bringing home only one food item at a time to feed its chick, and this renders food items smaller than fish energetically unsuitable for raising the chick. (Elliott et al. 2009). The main diet of nestlings in Canadian Arctic is arctic cod, but also capelin (*Mallotus villosus*) and sculpin (*Triglops*, *Gymnocanthus*, *Myoxocephalus* spp.) (Elliott et al. 2009). In contrast to the little auk and the thick-billed murre, the common eider is exclusively a benthic feeder, targeting mussels, crustaceans, and polychaetes at water depths often below 10 m (Merkel et al. 2007). In the spring, the eider needs open water around the small islands and skerries on which they breed to ensure that foxes are excluded. After laying the eggs, only the female attends the nest, and neither the female nor the chicks feed during the brooding period. This leaves a much smaller nutrient imprint in the colonies, but as males and non-breeding eiders also spend time in the colonies, some nutrients are deposited on land.

Analysis of the NOW food web, using stable isotopes, has further demonstrated that the three seabirds investigated here are linked to the marine ecosystem in different ways (Hobson et al. 2002). Thus, each species may display a different degree of dependence on the particular ecological conditions of the polynya, which is in part reflected by the distribution around Greenland and the wider Arctic. Polynyas are characterised by sustained periods (spring through summer) of open water and relatively high levels of primary production, from which all seabirds potentially benefit. However, whilst the common eider and the thick-billed murre are found in many areas around Greenland, large little auk colonies are found solely in relation to productive polynyas (NOW and Scoresbysund).

Here, we set out to elucidate the history of the seabird populations in the NOW area. Given the dependence of the little auk on the polynya, it may be possible to use information on the past population dynamics to provide insights into the history of the polynya itself. To do this, we used palaeoecological methods to investigate the history of seabird colonies on the east side of the NOW. The key questions were: (1) How long have the colonies been present? (2) Is there any evidence of variation over time? And (3) how do the arrival times and any variation in abundance over time relate to climatic variation and the history of human settlement in the region?

## Materials and methods

### Field methods

Fieldwork was undertaken in the summers of 2014 and 2015 with sedimentary archives collected from a lake and peat deposits within seabird colonies.

#### Core collection

A sediment core was extracted from a lake within a large little auk colony on Qeqertaq (Salve Ø) (Fig. 1). Lake coring was carried out using a highly portable percussion corer, which can be operated from a single boat (Chambers and Cameron 2001). Details of core locations, length, and depth of water at the coring site are given in Table 1. At Qeqertaq, rafting ice prevented access to the deepest area of the lake (24 m) and so a relatively deep flat-bottomed area (17 m) was cored. Cores from peat deposits were extracted at sites within a) little auk colonies at Qoororsuaq (SD1), Annikitsoq (GL3), and Kuukkat (RF1); b) from eider duck colonies at Iterlassuup Qeqertaarsui (TSB2) and Booth Sund (BS1) and c) below a large thick-billed murre colony on Appat (Saunders Ø) (Fig. 1). The peat cores were largely permafrost and collected using an SIPRE corer (Terasmae 1963) driven by an STIHL BT-121 two-stroke engine (details of the core location and depth are given in Table 1). At Appat and Annikitsoq, where there was a significant development of high-centre ice-wedge polygons, the centre of a polygon was selected for coring. In all other cases, the flattest area available, furthest away from the boulder fields and cliffs, was selected for coring to reduce the likelihood of (a) slumping and (b) large numbers of stones/rocks in the sample, respectively.

**Table 1:** Lake and peat cores locations and detail

Lake cores				
Code	Location	Core length (m)	Water depth (m)	Point of transition
Salva Ø, 04/08/2014				
NOW5a	76.044214	1.46	17	1.20
	− 65.984154			

Peat cores				
Code	Location	Material	Total depth below surface [cm b.s.]	Remarks
Three Sister Bees, 24/07/15				
TSB-2	76.76524	Pits and core	0–112	Active layer and permafrost (3 subprofiles)
	− 70.26229			
Booth Sund, 25/07/15				
BS-1	76.92206	Exposure	0–75	Active layer
	− 70.080611			
Saunders Island, 27/07/2015				
SI-1	76.56908	Pit and core	0–197	Active layer and permafrost
	− 70.04099			
Søkongedalen, 28/07/15				
SD-1	76.26716	Pit and core	0–97	Active layer and permafrost
	− 68.97227			
Annikitsoq 31/07/15				
GL-3	76.03288	Pit and core	0–320	Active layer and permafrost
	− 67.61811			
Robertson Fjord, 07/08/15				
RF-1	77.74599	Pit and core	0–99	Active layer and permafrost
	− 70.42283			

### Laboratory methods

#### Core dating

**Fig. 2:**
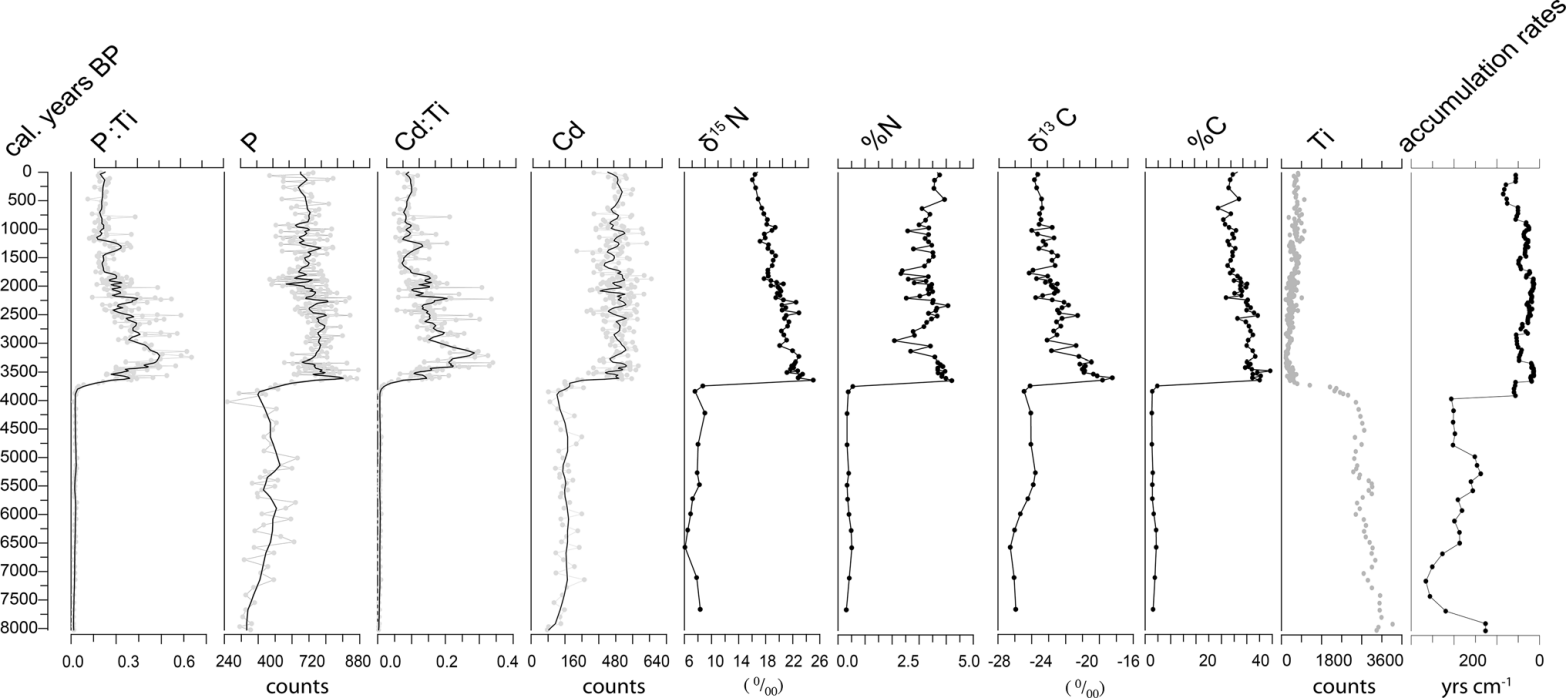
The NOW5a lake core from Qeqertaq (Salve Ø), selected indicators likely to be influenced by the presence of seabirds

Samples for radiocarbon dating were selected from the cores to provide either a reliable chronology of the entire sequence or, in the case of some of the peat cores, to establish the oldest age of the peat development. This estimate of the date peat began to form at the site is assumed to be the date of arrival of the bird colony. In the lake core, there was a clear transition in a range of indicators marking the point of bird arrival (Fig. 2). Above this change point, there were abundant terrestrial mosses from the families of the *Splachnacea* (dung mosses), *Polytrichacaea*, *Pottiaceae,* and from the genus *Distichium* (from the family *Ditrichacea*), which were sampled for ^14^C dating. Below the transition, terrestrial macrofossils were rare or absent, and thus, bulk sediment samples (humic acid extraction) were used for radiocarbon dating. Use of bulk samples can be problematic as it can give anomalously old ages, if old carbon has been incorporated into the system (Olsen et al. 2012). For the permafrost peat samples, organic matter free of shell material, likely of marine origin, was selected for dating. A full list of samples analysed and used to derive age models for the lake and peat cores is given in Table 2. The samples were dated at the Aarhus AMS ^14^C Centre at Aarhus University (AARAMS). The radiocarbon ages of the samples from the lake and the six peat cores were converted into calendar years using the IntCal13 calibration curve (Reimer et al. 2016). Age models for the cores were calculated using the R routine “Bacon”, a Bayesian age-depth modelling approach (Blaauw and Christen 2011). Ages reported and used in the figures are median modelled ages in cal. years bp (before present),^1^ with the minimum and the maximum of the 95% probability intervals are given in the text and in Table 2.

**Table 2:** Description of samples dated, ^14^C ages and modelled ages in cal. years bp from lakes and peat cores in the NOW, N.W. Greenland

Core code	Type	Depth	Material	Lab No	Age	Modelled dates	
						Modelled median age	95% probability intervals
					(^14^C bp)	(cal. yr bp)	(cal. yr bp)
Little Auk							
Qeqertaq (Salva Ø)							
NOW5a	Lake	14.5	Terrestrial macrofossil	AAR24215	943 ± 29	840	744 to 934
NOW 5	Lake	28.5	Terrestrial macrofossil	AAR24216	1337 ± 31	1281	1178 to 1352
NOW5a	Lake	42.5	Terrestrial macrofossil	AAR24217	1942 ± 54	1782	1599 to 1946
NOW5a	Lake	56.5	Terrestrial macrofossil	AAR24218	2029 ± 28	2025	1929 to 2152
NOW5a	Lake	70.5	Terrestrial macrofossil	AAR24219	2261 ± 31	2282	2159 to 2393
NOW5a	Lake	84.5	Terrestrial macrofossil	AAR24220	2519 ± 31	2635	2455 to 2769
NOW5a	Lake	98.5	Terrestrial macrofossil	AAR24221	3105 ± 31	3256	3050 to 3391
NOW5a	Lake	114.5	Terrestrial macrofossil	AAR29954	3265 ± 41	3575	3425 to 3771
NOW5a	Lake	122.5	Bulk	AAR24730	3695 ± 28	3846	3753 to 4037
NOW5a	Lake	133.5	Bulk	AAR24731	5184 ± 31	5308	4883 to 5799
NOW5a	Lake	144.5	Bulk	AAR24732	7283 ± 30	7819	7054 to 8184
				Transition point		3645	3510 to 3812
Annikitsoq							
GL3	Permafrost	5–10	Peat	AAR24684	652 ± 32	703.3	643.5 to 788
GL3	Permafrost	48–52	Peat	AAR24685	2474 ± 26	2524.2	2439.7 to 2608.3
GL3	Permafrost	87–91	Peat	AAR24686	2993 ± 33	3145.4	3076.4 to 3213.7
GL3	Permafrost	127–131	Peat	AAR24687	3365 ± 28	3536.1	3503 to 3587.5
GL3	Permafrost	149–153	Peat	AAR24688	3417 ± 27	3658	3622.5 to 3695.1
GL3	Permafrost	191–195	Peat	AAR24689	3553 ± 41	3821.9	3784.3 to 3872.4
GL3	Permafrost	227–231	Peat	AAR24709	3605 ± 26	3963	3930 to 3994
GL3	Permafrost	248–252	Peat	AAR24690	3720 ± 31	4046.1	4021.2 to 4083.6
GL3	Permafrost	267–271	Peat	AAR24691	3568 ± 39	4112.5	4086.3 to 4170.2
GL3	Permafrost	300–305	Peat	AAR24692	3802 ± 41	4305.1	4254.2 to 4367.7
GL3	Permafrost	316–320	Peat	AAR24693	3855 ± 30	4409.9	4365.3 to 4444.2
Kuukkat (Robertson’s fjord)							
RF1	Permafrost	5–10	Peat	AAR24694	Pre 1960	9.8	− 32.9 to 77.7
RF1	Permafrost	25–29	Peat	AAR24695	250 ± 27	344.8	293 to 418.4
RF1	Permafrost	49–54	Peat	AAR24696	794 ± 25	769.8	738.8 to 816.5
RF1	Permafrost	75–80	Peat	AAR24697	1537 ± 34	1520.5	1448.1 to 1576.8
RF1	Permafrost	95–99	Peat	AAR24698	2223 ± 34	2247.3	2176.5 to 2317.8
Qoororsuaq (Søkongdale)							
SD1	Permafrost	10–12	Peat	AAR24705	225 ± 30	229.1	165.9 to 345.2
SD1	Permafrost	48–52	Peat	AAR24706	2074 ± 26	2027.7	1725 to 2116
SD1	Permafrost	70–74	Peat	AAR24707	2553 ± 28	2231.2	2043 to 2379.9
SD1	Permafrost	70–74	Peat	AAR24707	2280 ± 27	2761.2	2706-2815
Thick-billed Murre							
Appat (Saunders Island)							
SI1	Permafrost	0–10	Peat	AAR24699	Pre 1960	51	− 19.1 to 167.8
SI1	Permafrost	42–46	Peat	AAR24700	1114 ± 27	1157.9	1084.5 to 1254.3
SI1	Permafrost	64–68	Peat	AAR24701	2912 ± 27	3133.6	3061.8 to 3221.2
SI1	Permafrost	103–107	Peat	AAR24702	4182 ± 26	4702.3	4624.5 to 4767.9
SI1	Permafrost	144–148	Peat	AAR24703	4530 ± 27	5185.1	5132.8 to 5246.8
SI1	Permafrost	191–195	Peat	AAR24704	4851 ± 27	5648.1	5613.1 to 5705
Common Eider							
Iterlassuup Qeqertaarsui (Three Sister Bess)							
TSB2	Permafrost	80–84	Peat	ARR25292	2891 ± 45	2954.3	2833.5 to 3079.8
TSB2	Permafrost	110–112	Peat	ARR24682	4804 ± 29	5486.6	5391.6 to 5558.7
Booth Sund							
BS1	Exposure	45–49	Peat	ARR25294	989 ± 34	970.4	897.6 to 1035.1
BS1	Exposure	74–75	Peat	ARR25293	2337 ± 35	2235.3	2115.5 to 2359.7

#### Peat core sampling

The frozen peat cores were first split at approx. – 8 °C and sectioned into 2–4 cm slices and freeze-dried (Zirbus Subliminator 3–4–5). The gravimetric ice content was measured as the weight difference between fresh and freeze-dried bulk sediment samples, and it is expressed as ice content in weight percentage (wt%). The samples were powdered using a Fritsch pulverisette 5-mill equipped with agate jars. To quantify the total contents of carbon (TC) and nitrogen (TN), each sample was prepared twice and measurements were carried out on an elementar vario EL III elemental analyser. About 5 mg of the sample were put into tin capsules, combined with a small amount of tungsten(VI) oxide to catalyze the full combustion of the sample in the varioEL. To calibrate the measurement, a set of calibration standards consisting of acetanilide, sucrose, and 30% EDTA was used. In addition, every 15 samples, a control sequence of 30% EDTA, 20% EDTA, 12% calcium carbonate, IVA33802150 (soil standard, C = 6.7%, N = 0.5%, S = 1.0%), and soil standard 1 (C = 3.5%, N = 0.216%) was measured. The accuracy of the measurement was ± 0.1% for nitrogen and ± 0.05% for carbon.

To differentiate the total organic carbon (TOC) content, the samples were measured using an elementar varioMAX C elemental analyser. The sample mass to use was calculated from the total carbon content, giving weights between 15 and 20 mg, which were filled into steel crucibles. 30% glutamate, pure glutamate, and 2:3 glutamate were used for calibration. The control sequence consisted of 2:3 glutamate, 10:40 glutamate, 5:45 glutamate, and 1:19 glutamate, repeated every 15 samples. The accuracy of the measurement was ± 0.1%. Subsequently, a ratio was calculated from TOC and TN, referred to as C/N.

#### Stable isotopes

Stable isotopes of C and N were analysed as they have been shown to provide evidence of the influence of marine derived nutrients from a range of sources in terrestrial and freshwater ecosystems, using both contemporary (González-Bergonzoni et al. 2017) and palaeoecological approaches (Finney et al. 2002; Michelutti et al. 2013). For the analysis of the stable isotopes of C and N in the peat cores, carbonate was first removed from the samples. About 2 g of each sample was transferred into 100 ml Erlenmeyer glass flasks, dosed with 20 ml 1.3 mol hydrochloric acid, and heated at 97.7 °C for 3 h. To get rid of the chloride ions as they would interfere with the isotope analysis, the flasks were repeatedly filled up with purified water and allowed to settle until the chloride content was < 500 ppm. To regain a dry state, the sample solution was then filtered under vacuum using GE Healthcare Life Sciences Whatman glass microfiber filters, dried at 50 °C and subsequently ground by hand before being transferred into plastic jars. Preparation for measurement was executed by placing the samples in tin capsules, where each target weight was calculated as 20/TOC. Stable carbon (*δ*^13^C) and nitrogen (*δ*^15^N) isotope analysis was undertaken using a Thermo Scientific Delta V Advantage Isotope Ratio MS supplemented with a Flash 2000 Organic Elemental Analyser using helium as a carrier gas. The accuracy was better than ± 0.15‰ for *δ*^13^C and ± 0.2‰ for *δ*^15^N.

In the lake core, samples were taken at 2 cm intervals, freeze-dried for 48 h, and ground into fine powder. Test samples were analysed to determine the appropriate mass of sample for analysis, which was 3 mg post-transition point and 15 mg pre-transition point. The samples were packed into tin cups and sent to UC Davies Stable Isotope Facilities, California, USA, where they were analysed following the standard procedures (see http://stableisotopefacility.ucdavis.edu). It was not necessary to pre-treat the samples to remove carbonates owing to the very low pH of the lake pH < 4.

#### Metal analysis ICP-MS

Metal concentrations in sediments have been used to track seabird influence on land and fresh waters in the previous studies (Outridge et al. 2016). Cadmium (Cd) and phosphorus (P) are more abundant in marine waters and become concentrated up the food web, and these elements have been used to track seabird populations in the previous studies (Wagner and Melles 2001; Bennike et al. 2007).

Samples of peat were analysed for trace element composition at the accredited trace element laboratory at Department of Bioscience, Aarhus University, in Roskilde, Denmark. Peat samples were dried and samples consisting of c. 0.1 g dry weight were microwave digested in Teflon bombs in 2 ml/2 ml Merck Suprapure HNO_3_/MilliQ water using an Anton Paar Multiwave 3000 oven.

Digestion solutions from peat were diluted with MilliQ water and analysed for 61 elements including phosphorus (P), titanium (Ti), and cadmium (Cd) (only P, Ti, and Cd are presented in this study) using an Agilent 7900 ICP-MS.

The analytical quality was checked by analysing blanks, duplicates, and a selection of Certified Reference Materials (CRM) along with the samples. For peat samples, the CRM included MESS-4, PACS-2, and BCR-482 (two marine sediments and a lichen, respectively). Detection limits for P, Ti, and Cd (determined as 3 SD on blank values) were 10, 0.06, and 0.0009 mg/kg dry weight, respectively.

#### Scanning XRF analysis

The scanning X-ray fluorescence is a non-destructive method of measuring metal concentration of sediment cores at potentially very high resolution, down to 0.1 mm scale. The lake sediment core was split along its length then placed in an ITRAX core scanner to obtain high-resolution pictures and measure micro-XRF. The XRF scans were made at the Aarhus University core-scanning facility with a molybdenum tube set at 30 kV and 30 mA with a dwell time of 4 s. Prior to analysis, the sediment surface was flattened and covered with a 4 mm ultralene film. A step size of 0.1 mm was selected to capture possible elemental variations even in small laminations.

## Results

### Core chronologies and markers of bird arrival

The radiocarbon samples and ^14^C dates from the peat and lake cores collected are given in Table 2, as are the calibrated modelled ages and the 95% confidence intervals as determined using the bayesian age depth modelling approach (Blaauw and Christen 2011) (Electronic Supplementary Material, Figs. S1–S7). Elemental composition, stable isotope ratios of C and N and selected geochemistry (Fig. 2) combined with ^14^C dating and modelling, estimate the arrival time of the little auk colonies at Qeqertaq (Salve Ø; NOW5a) to 3650 cal. years bp, with the 95% probability intervals ranging from 3500 to 3800 cal. years bp. For the peat cores, dating the base of the core provides the estimate of arrival time, since the formation of the peat is dependent on the marine derived nutrients (MDN) supplied by the birds (Bennike et al. 2007) as there is no peat formation in the region in the absence of current or past bird influence (Mosbech et al. 2018). At Annikitsoq (GL3), the median estimate of little auk arrival was 4400 cal. years bp, with the 95% probability intervals ranging from 4360 to 4440 cal. years bp. At the little auk colonies at Qoororsuaq and Kuukkat, the median basal dates of the peat cores were 2760 cal. years bp (95% probability interval of 2710–2810) and 2250 cal. years bp (95% probability interval of 2180–2320), respectively.

Based on the basal dates of the peat cores, and the age-depth models (albeit based on few samples), we estimate the arrival of the common eider at Iterlassuup Qeqertaarsui (Three Sister Bess) to be 5490 (95% probability interval of 5400–5560) and at Booth Sund 2240 (95% probability interval of 2100–2400) cal. years bp. The basal date of the peat deposit beneath the thick-billed murre colony at Appat (Saunders Ø) suggests an arrival date of 5650 (95% probability interval of 5600–5700) cal. years bp.

### Sediment accumulation rates

**Fig. 3:**
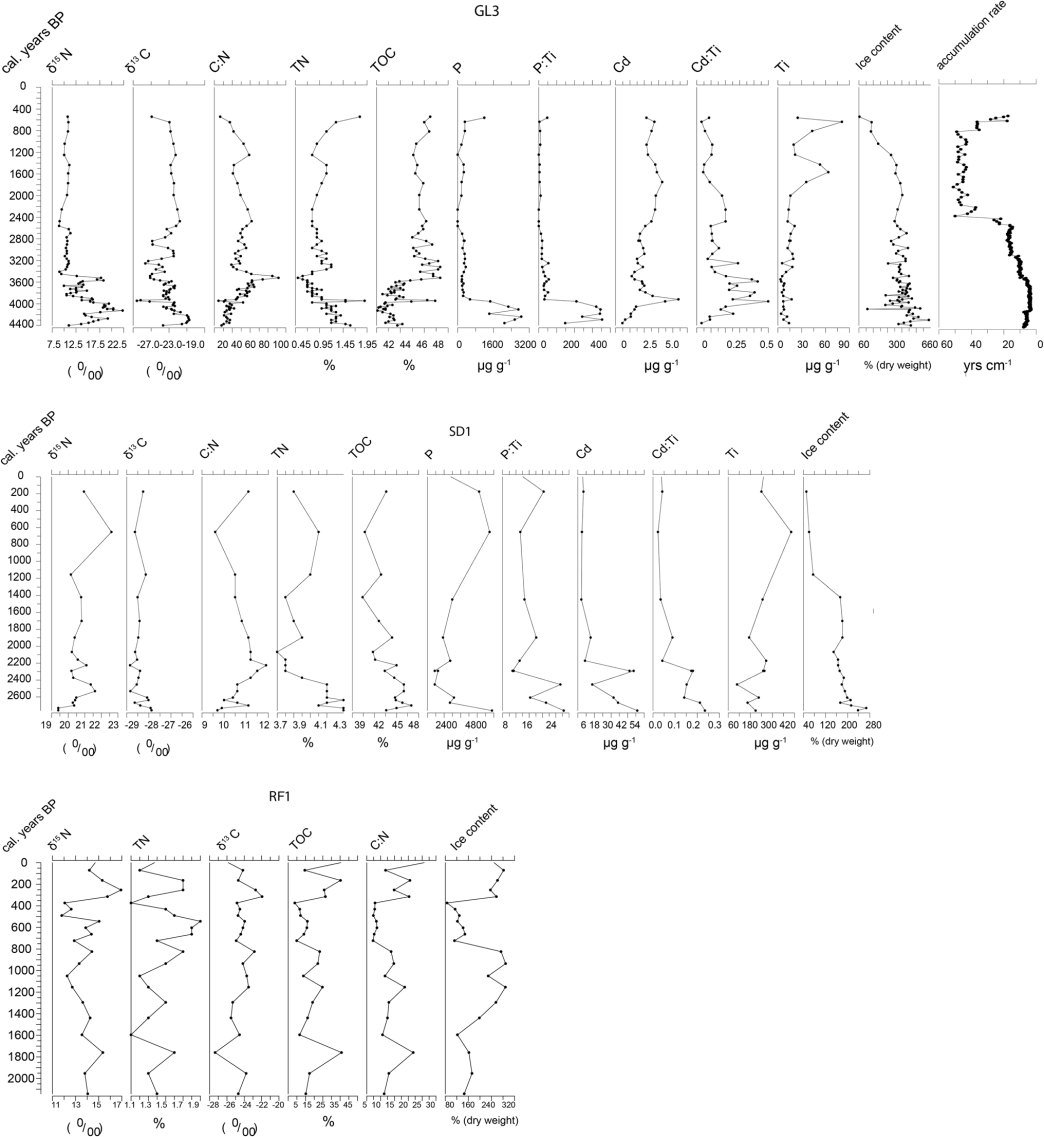

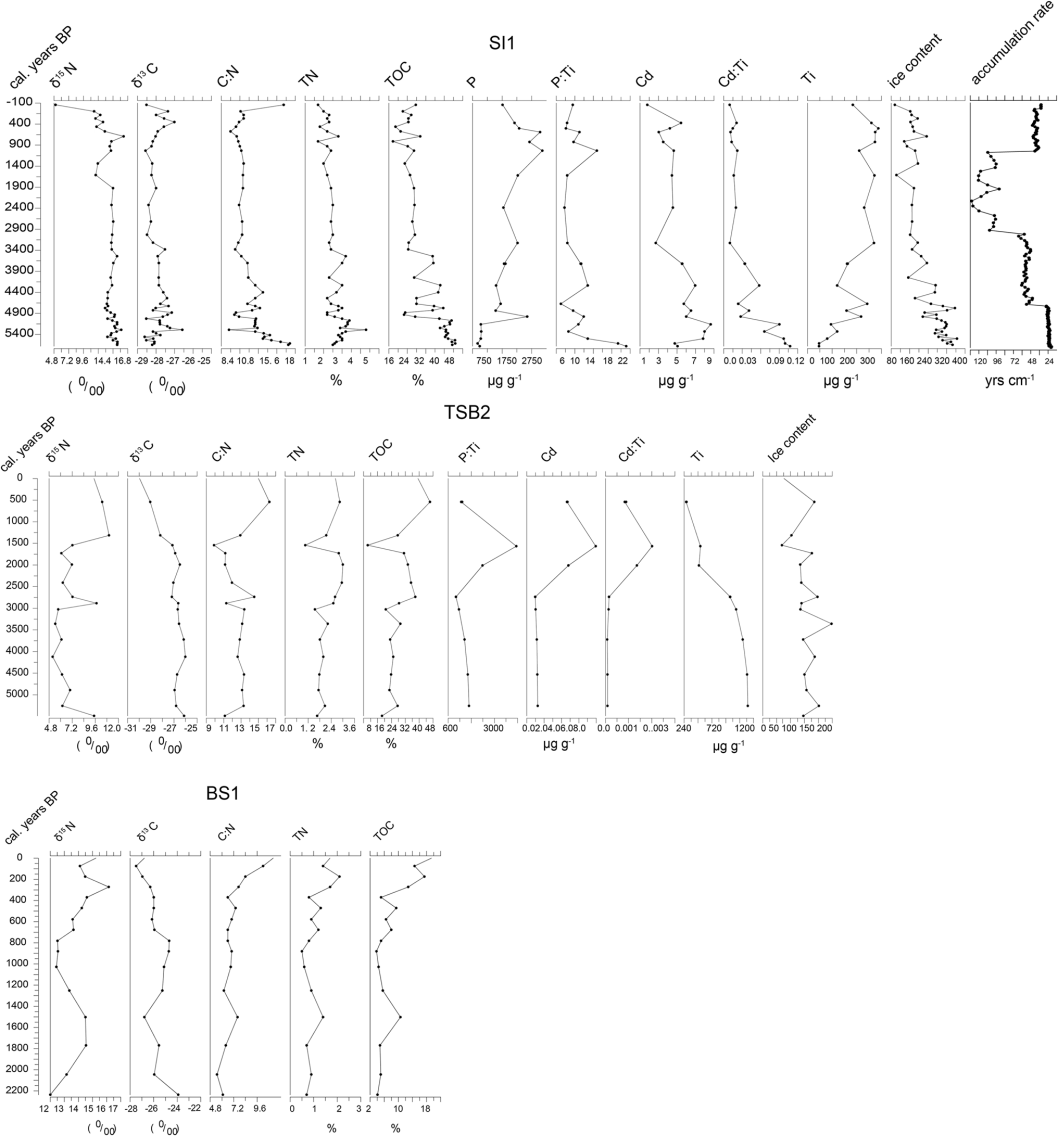
**a** Selected proxies of seabird activity from the Little auk peat core records for GL3 Annikitisoq, SD1 Qoororsuaq (Søkongdale), and RF1 Kuukkat (Robertson’s fjord). **b** Selected proxies of seabird activity for thick-billed murres from core SI1 Appat (Saunders Ø) and eider duck records from the peat cores TSB2, Iterlassuup Qeqertaarsui, and BS1 at Booth Sound

There was considerable variation in sediment accumulation rates, both between the localities and within individual records. At Appat (Saunders Ø), there was a very fast accumulation rate between 6000 and 4500 cal. years bp (Fig. 3b), and then a slight slowing down of accumulation around 4500, which lasted to 2900 cal. years bp. There followed a further large decrease to a very low accumulation rate of around 90 yrs/cm from 2700 to around 1000 cal. years bp, where there was a very marked increase in accumulation rates.

At the common eider colonies, the resolution of ^14^C sampling was too low to make the determination of accumulation rates possible. For the little auk colonies, however, the peat cores provide some information. At Annikitisoq (Fig. 3a), the record is divided in two around 2400 cal. years bp with high accumulation rates before this point, and low accumulation rates after. There was also indication of some change in the period 4400–2400 cal. years bp, with faster accumulation around 4200 cal. years bp which was then stable with some slight decreases in rate before the vary large decrease in accumulation rate at 2400 cal. years bp. At Kuukkat and Qoororsuaq (SD1), the dating models are based on too few samples to give meaningful accumulation rates.

The accumulation rates of the NOW5a core (Fig. 2) show considerable variation with a very large increase at the arrival of the little auk at 3650 cal. years bp. There is then a reduction centred around 3000 cal. years bp with higher accumulation rates at 2700 cal. years bp and then around again at circa 1900 cal. years bp.

### The lake core record of little auk presence and abundance at Qeqertaq (Salve Ø)

Figure 2 illustrates selected parameters from the Qeqertaq (Salve Ø—NOW5a) lake core likely to reflect the presence of seabirds, including ratios of XRF generated cadmium (Cd), phosphorus (P), and titanium (Ti) data, along with *δ*^15^N, *δ*^13^C, %C, and %N of the sediments. The magnitude of change reflected by the sedimentary record at the time of bird arrival was unprecedented and likely changed all aspects of the ecosystem structure and function as was observed present day (González-Bergonzoni et al. 2017) (Fig. 2). Organic carbon content increased from around 5% C content to over 40%, demonstrating a large change in the primary productivity of the lake. The shift in *δ*^15^N values reflects wholesale alteration of the nutrient sources and provides an unequivocal marker of the input of MDN (González-Bergonzoni et al. 2017) which marks the point of arrival of the little auk colony. Compared with the transition associated with the arrival of the little auk in the catchment, the variation afterwards is relatively small. The ratios of both P and Cd to Ti show a peak centred around 3250 cal. years bp covering around 250 years. There follows a relatively sharp decline in both P/Ti and Cd/Ti to the year 3000 cal. years bp; this coincides with a fall in the %N composition. From 3000 cal. years bp to 2300 cal. years bp, there is a period where the loess smoothers of the Cd/Ti and P/Ti vary little, but end in a peak at circa 2300 cal. years bp. Whilst the loess smoothers do not vary a great deal over this time period, there is a relatively large amount of variation around the smoother. Over this time period of 3000 cal. years bp to 2300 cal. Years, bp *δ*^15^N and *δ*^13^C were stable and relatively high, whereas % N gradually increases, but all three came to a peak around 2400–2300 cal. years bp. Thereafter, all these indicators show a marked fall ca. 2100 cal. years bp for a relatively short period, in the case of *δ*^13^C and %N. In general, after 2000 cal. years, bp values of all the little auk indicators become more stable with fewer high values and a general decline to consistently low levels post-1900 cal. years bp until 1500 cal. years when there is a rise in Cd/Ti, P/Ti, *δ*^13^C, and % N around bp. Thereafter, there is a decline at around 1200 cal. years bp and then relative stability with a slight upward trend to the present.

### Peat cores

#### Annikitsoq (GL3), Qoororsuaq (SD1), and Kuukkat (RF1) and the little auk

Figure 3a shows peat core profiles for the three little auk colonies sampled. The core from Annikitisoq (GL3) covers the longest time period with a basal date, reflecting the little auk colonisation, of 4400 cal. years bp. From the start of peat formation to ca. 3500 cal. years bp, there was a period of relatively large variation in *δ*^15^N with a slow increase to around 4100 then a gradual decline to 3800 where it remained stable and relatively low until a sharp and brief increase at around 3600 cal. years bp, followed by a sharp fall to a low at 3400 cal. years bp. This early period of the core was also characterised by a gradual decline in N from 4400 cal. years bp to very low levels at around 3500 cal. years bp; during this time, TOC was relatively stable, and thus, the C:N ratio gradually increased over this time peaking at exceptionally high values of nearly 100 at around 3500 cal. years bp when N was at its lowest concentration. As stated, the records of P, Cd, and their ratios with Ti may provide some information on variation in bird influence at the site (Wagner and Melles 2001). Phosphorus was initially extremely high and matched the variation in the *δ*^15^N record until circa 3800 cal. years bp, where there was a large decline in *δ*^15^N, *δ*^13^C and P but a large increase in Cd and an increase in Ti, TOC and TN. This large excursion around 3900 cal. years bp may not be connected to inputs from the bird community as the MDN would also cause the P and *δ*^15^N to rise—which did not occur. Apart from this large excursion, there was good agreement between P, Cd, and their ratios Ti and *δ*^15^N in the early record with declines around 3800 cal. years bp. There followed a rise in *δ*^15^N at circa 3600 cal. years which was reflected in Cd and Cd/Ti ratios, and in the P/Ti ratios, the latter is difficult to see in Fig. 3a as the previous levels were so high. These previously very high values of P at the bottom of the record, associated with very high accumulation rates, suggest abundant seabirds for at least 400 years. This early period of large variation was followed by a period of stable values from 3500 cal. years bp to 2500 cal. years bp at which point *δ*^15^N fell to its lowest value in the record. At this time, P and P/Ti fell to zero before rising slightly at 2200 cal. years bp, thereafter, remaining rather low and falling to zero again around 1200 cal. years bp. In contrast at 2500 cal. Years, bp Cd/Ti rose a little. At Annikitisoq, there was what appears to have been an input of terrestrial minerogenic material 1500 cal. years bp as a number of indicators, such as P and Ti, both rise sharply, whereas Cd did not and there are no dramatic changes in any of the other indicators (*δ*^15^N, TN, and *δ*^13^C).

The records from Qoororsuaq (SD1) and Kuukkat (RF1) both cover a much shorter time period. At Qoororsuaq, the resolution of sampling of C and N, isotopes, and geochemistry towards the top of the core is lower, and the age-depth models are based on fewer samples and may thus lack the temporal resolution to accurately identify points of variation. However, SD1 shows some agreement with the longer record from Annikitsoq in that the data suggest the greatest variability between 2700 and 2000 cal. years bp, while TN values suggest a decline in bird input from around 2200 to 2000. The data on Cd and P are not as clear, suggesting elevated inputs between 2400 and 2200 cal. years bp. At Kuukkat, the variation in the parameters measured is rather low, suggesting a rather stable population over time, but, in the absence of geochemical data, this is less certain.

#### Appat (SI1) and the thick-billed murre

In comparison with the records from the little auk colonies, the peat core from the thick-billed murre colony at Appat (Saunders Ø) shows less variability over time. The record indicates changes in peat productivity/accumulation rates with high TOC content at the base of the core around 5000 cal. years bp, where the climate was likely warm (Briner et al. 2016; Lecavalier et al. 2017). Over the initial period of high accumulation to around 4500 cal. years bp, *δ*^13^C and *δ*^15^N show a large degree of variation; TOC and TN also show some variation with rising values, perhaps, suggesting increased marine inputs. Post 5000 cal. years bp, TOC and TN start to vary, initiated by a sharp drop in TOC, which would indicate a decline in productivity followed by marked variation in TOC content until around 3400 cal. years bp. In general, however, the record indicates the consistent presence of seabirds with little change. However, the decline in *δ*^15^N at the top of the core is notable.

#### Booth Sund (BS1) Iterlassuup Qeqertaarsui (TSB2) and the common eider

The two records from common eider colonies cover very different time spans. At Booth Sund, the record covers around 2200 years and shows very little variation, though there are increases in TN, TOC, and *δ*^15^N over the last 800 years, and, perhaps, more markedly over the last 400 years. The record from Iterlassuup Qeqertaarsui (Three Sister Bess; TSB2) is much longer, covering around 5500 years. With a couple of exceptions, stable isotope values and geochemistry also indicate a relatively stable record in this case. In contrast with the other records, the accumulation rate was relatively low soon after the start of the record, with a large reduction around 3000 cal. years bp. This fall was coincident with a rise in *δ*^15^N and to a lesser extent a rise in TN and TOC. After this period, post-2700 cal. years bp, there was an increase in the elements associated with the transport of MDN (P and Cd and their ratios with Ti).

### Synthesis of data on change in little auk populations

The oldest recorded date of arrival of the little auk found here was the 4400 cal. years bp at Annikitsoq, and the dates of arrival at the other sites are 3600 cal. years bp at Qeqertaq (Salve Ø), 2700 cal. bp at Qoororsuaq, and 2200 cal. years bp at Kuukkat (Fig. 1). Synthesising the data from the different indicators of little auk abundance (*δ*^15^N, *δ*^13^C in the lake core, Cd/Ti and P/Ti) from the peat core at Annikitsoq suggests that, initially, variation in bird numbers was high with a fall from around 4000 to 3800 cal. years bp and then a rise again at 3600 cal. years bp. This latter rise corresponds to the date of colony formation at Qeqertaq. After 3500 cal. years bp at Annikitsoq, the *δ*^15^N variation does not suggest large amounts of variation in bird numbers; however, the large shift in accumulation rate at 2500 cal. years bp could indicate a large decline in nutrient input, which coincided with P concentrations falling to zero. The lake core from Qeqertaq provides much greater temporal resolution than the peat cores and this record indicates relatively high bird abundance until around 3000 cal. years bp followed by relatively large variability in the input of MDN (as indicated *δ*^15^N, *δ*^13^C, Cd/Ti, and P/Ti) in the period from 3000 to 2000 cal. years bp, but with a peak centred around 2200 cal. years bp. This latter date correspond well with the arrival time of little auks at Kuukkat.

The peat core from Annikitsoq suggests a decline in bird numbers around 3800 cal. years bp, and the P and P/Ti record may suggest a complete absence from 2500 to 2200 cal. bp and low numbers from 1700 to 1200 cal. years bp. The higher resolution record from the lake at Qeqertaq shows much greater variation than the peat cores. P, Cd, and their Ti ratios indicate a low input of MDN at Qeqertaq from around 1700 cal. years bp, which persisted until 1500 cal. years bp. After this point, there were no extended periods of low P or Cd, suggesting that the little auks were consistently present.

## Discussion

Identification of the point of arrival of seabirds in a particular catchment is relatively straightforward as the transport of marine-derived nutrients (MDN) transforms the landscape (González-Bergonzoni et al. 2017; Mosbech et al. 2018). The estimated time of arrival can be determined by dating basal samples from peat cores, or the point of marked increases of *δ*^15^N in lake sediments. Values of *δ*^15^N in organic matter in lake sediments not affected by seabirds from the High Arctic are generally not higher than 3‰ and seldom rise higher 4–5‰ (e.g. Janbu et al. 2011; Perren et al. 2012). A rise in *δ*^15^N of 2–3‰ *δ*^15^N has been used in other studies to track millennial scale change in sockeye salmon population in Alaska (Finney et al. 2002) and increases to levels similar to those reported here, up to and > 20‰, have been used to track human influence on fresh waters, via transport of MDN, on the Canadian side of the NOW (Michelutti et al. 2013). Furthermore, there is an almost total absence of peat accumulation in the NOW region outside bird colonies (Mosbech et al. 2018), and in addition, the *δ*^15^N values of the peat cores, though variable between sites, are much higher than values reported for non-bird driven peat accumulation (Skrzypek et al. 2008). Thus, the combination of data presented here provides an unequivocal marker of bird arrival. The evidence indicates that the earliest arrival of the little auk in the NOW region was around 4400 cal. years bp, whereas the thick-billed murre and the common eider have been present for at least 1500 years longer. The three species discussed here breed in completely different habitats and landscape settings, so there is no possibility of a change in bird community at a particular site.

**Fig. 4:**
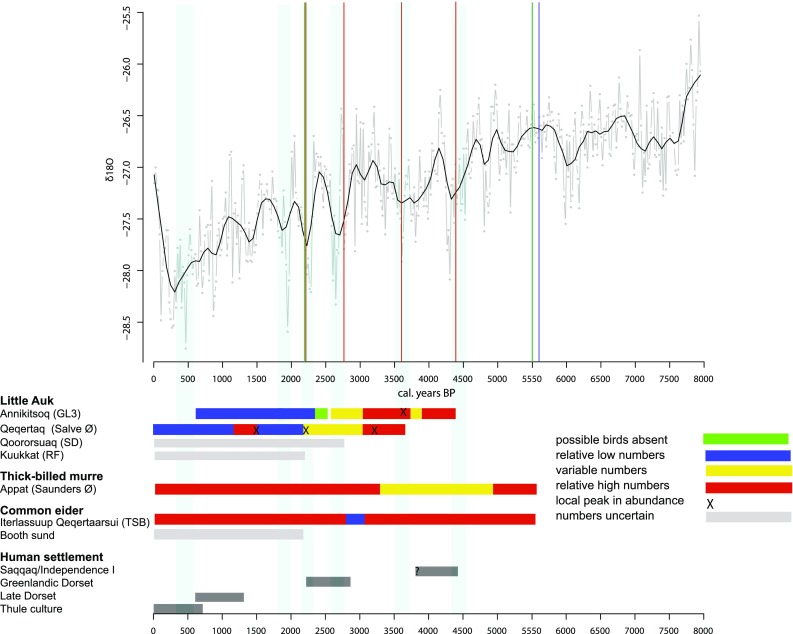
Ice core *δ*^18^O from Aggasiz which reflects temperature change over the last 8000 years. The vertical lines indicate the arrival points of the various bird colonies. Red for little auk, blue is the thick-billed murre, and green the eider. The pale blue vertical bands mark periods of colder air temperature as inferred form the ice core isotope record. Below there is summarised information on the inferred variation in bird numbers at the sampled colonies and the history of human habitation of the NOW area derived from archaeological records

A striking feature of the arrival times of the little auk at the sites across the NOW, compared with the thick-billed murre and the eider duck, is the correspondence with periods of cooler climate, as reflected by the oxygen isotope data from the Aggasiz ice core (Fig. 4) (Vinther et al. 2009). Though, it should be noted that not all cool periods correspond to an arrival event, for example the period of 1900 cal. years bp, but then, only four of more than a hundred little auk colonies in the region were dated (Boertmann and Mosbech 1998). In contrast, the earliest record of the arrival of the thick-billed murre and the common eider correspond with a period of warmer climate around 5500 cal. years bp (Fig. 4). The common eider is a shallow water, benthic feeder, which needs ice-free (and thus fox free) conditions around its breeding series. The thick-billed murre needs fish to feed its chick—it is only capable of bringing one food item home at a time, and thus needs large food items for foraging to be feasible. In particular, the common eider, but also to some degree the thick-billed murre, have an extensive breeding distribution around Greenland, and seem capable of inhabiting many different habitats. In contrast, breeding colonies of little auks in Greenland are almost exclusively found in proximity to High Arctic polynyas, first and foremost the NOW but also in connection with the polynya at the mouth of Scoresby sund. To complete its breeding cycle, the little auk requires a sustained population of large copepods (*C. hyperboreus* and *C. glacialis*) in the upper 50 m of the water column during chick rearing in July/August, and presumably also in May/June during the early stages of the breeding season. This is provided only by a high Arctic marine ecosystem with open water and sustained primary production throughout spring and summer, combined with limited competition from fish predation on the copepods. In Greenland today, these conditions are exclusively found around polynyas, and thus, it is exceedingly unlikely that little auks would be present in large enough numbers to transform the landscape in the absence of a polynya. This means that our oldest date of a little auk colony 4400 cal. years bp may be seen as a minimum age of the NOW polynya ecosystem. It may well be possible that the NOW polynya first formed at this point in time, which corresponds to the end of the mid-Holocene Thermal Maxima and the onset of the cooling associated with the Neoglacial period (Vinther et al. 2009; Briner et al. 2016).

The idea that the NOW polynya first developed at a time of climate cooling is in good accord with the existing knowledge on the formation of the polynya. The formation of the NOW polynya primarily rests on the establishment of an arch or bridge of fast ice across Nares Strait in the southern part of Kane Basin, which blocks the southerly flow of drift ice from the Polar Sea (Melling et al. 2010). South of the ice arc in Smith Sound/Northern Baffin Bay, new ice forms on the sea surface but is continuously blown southwards by the prevailing northerly winds and currents. This creates the open water area, and at the same time, this ‘ice factory’ promotes brine formation and downwelling/upwelling, which seeds the system with nutrients, perhaps, supplemented by the West Greenland Current. The formation of the ice bridge may be a classic tipping point (Jeppesen et al. 2018), where a small change in temperature results in a large alteration of the surrounding ecosystems. In this case, perhaps, somewhat counter intuitively, the open water area of the NOW polynya is reliant on colder temperatures. In addition, colder air temperatures would result in a larger amount of new ice production in the ‘ice factory’, more brine formation, and greater circulation of the system. The fact that the little auk population arrived in and, perhaps, expanded during colder periods may indicate a link between the success of the little auk and climate. Thus, we speculate that a colder climate overall results in a stronger and more consistent ice bridge, more open water, more nutrients, increased primary production, and larger Calanus populations—all beneficial to the little auk. Lower temperatures may further imply reduced competition from fish predation on the large Calanus spp., as fish species like Capelin (*Malotus vilosus*) may be metabolically limited and retract their distribution southwards during colder periods (Rose 2005).

Caution must be exercised in interpreting the sediment record from the lake at Qeqertaq and also the peat cores, though their lower temporal resolution is already acknowledged. Whilst the dating of the arrival of the little auk is relatively straightforward, inferring changes in abundance can be more difficult. This is because the nitrogen in the system stored in the lake itself but also in the extensive peat developed in the catchment can be continually recycled, and thus, the ‘memory’ of past bird populations may be preserved in the *δ*^15^N signal, reducing its utility as an indicator of bird abundance. This may be particularly true for the lake at Qeqertaq where there is no other source of water input to flush the system in the event that birds were absent. The catchments soils also provide a large buffer/smoother for the lake as nitrogen enriched in *δ*^15^N will continue to leach from the soils into the lake for decades to centuries even in the absence of a bird colony. However, the combination of a number of indicators of bird abundance, in this case *δ*^15^N, *δ*^13^C (which in the lake reflect inputs of marine derived carbon), and Cd and P appear to provide a relatively robust semi-quantitative means of tracking relative change in bird abundance.

Cadmium and P data have been used in other studies (Wagner and Melles 2001) to infer change in little auk populations as both are abundant in little auk droppings. Both these elements are more abundant in sea water and are concentrated by marine zooplankton, an important food source of the little auk. Here, we also used the ratio of these elements to Ti. Ti content of seabird guano is low, whereas Ti is an indicator of input from catchment erosion which, dependent on geology, may supply both Cd and P. Thus, the ratios of Cd and P to Ti are more likely to reflect the input of the little auk, with any catchment input removed. Cd and P both suffer, to some degree, the same problem as *δ*^15^N as they can be stored in catchment soils and can also be mobile in the sediment cores. However, both seem to provide some evidence for variations in little auk numbers. It is, however, difficult to discuss absolute numbers with any certainty, but the records suggest that there has been a relatively large variation in little auk numbers since their arrival circa 4400 cal. years bp. The records, the peat core from Annikitisoq and the lake core from Qeqertaq, do not entirely agree, but combining the records, taking into account *δ*^15^N, *δ*^13^C, Cd/Ti, and P/Ti the data from Annikitisoq suggest that there was a decline in bird numbers around 4000 to around 3600–3700 cal. years bp, whereupon numbers rose again. This latter date is the point at which the colony was established at Qeqertaq (Fig. 2). The very high accumulation rates of the peat at Annikitisoq (GL3) suggest that there were abundant nutrients until at least 3500 cal. years bp, at which point nitrogen content of the peat cores was extremely low. It is difficult to interpret the records for the next 1000 or so years, but the general indication is a period of relative stability with some increases in bird influence around 3200 and then a decline around 3000 cal. years bp. At around 2500 cal. years bp, a number of the records indicate change, at Annikitisoq accumulation rate falls, P and P/Ti fall to zero, although Cd is still present. At Qeqertaq, P, Cd, and their ratios with Ti fall also fall around 2500 cal. years bp, but the isotope records and % N do not indicate change. This may be due to a mismatch in temporal resolution of the observations. P, Cd, and Ti are XRF data measured at higher temporal resolution (50 mm), whereas the isotope data are measured every 2 cm, so large short-term variation may be missed by the isotope data. The data do, however, agree that there was a peak at 2200 cal. years bp followed by a fall in indicators of bird input at Qeqertaq. The peak at 2200 cal. years bp corresponds with the arrival time of the colony at Kuukkat. Post-2200 cal. years bp, there follows a period of instability with sharp increases and falls in these key elements with a notable low point in P, Cd, and their ratios with Ti post 2000 cal. years bp, which remained low until 1500 cal. years bp.

The changes in bird numbers suggested by the data must be treated with some caution, as there is a relatively large degree of uncertainty associated with inferred changes. However, if we try and summarise the change in little auk numbers and by inference the polynya size and productivity the data suggest a more productive polynya at 4400, 3600, 2700, 2200, and around 1500 cal. years bp and a less productive or smaller polynya at 3800, 2500, and 2100–1550 cal. years bp.

### Little auks, polynya strength, and human settlement: Possible correlations

The synthesis of the data presented here has led us to the reasonable hypothesis that the establishment of little auk colonies and their fluctuating abundance over time may reflect the formation and changing conditions of the NOW polynya. It is interesting to examine how these inferred fluctuations in the size or ‘strength’ of the polynya are related to the known history of human presence/absence in the region.

Evidence from radiocarbon dates of the first Arctic Small Tool tradition societies in the Eastern Arctic (Saqqaq/Independence I) was recently subjected to thorough analysis (Grønnow 2017). It was concluded that the NOW (and the rest of Greenland) was settled by pioneer societies as part of a remarkably fast, initial spread of humans from the Western/Central Canadian Arctic into Eastern and High Arctic Canada and Greenland sometime during the period of 4420–4290 cal. years bp. Thus, there is a remarkable agreement in the timing of our oldest date of little auk colonies and the arrival of humans in the area. The following human abandonment of the High Arctic around 3800 cal. years bp (Grønnow and Jensen 2003) takes place during a period of low or instable occurrence of little auks, i.e. a ‘weak’ polynya condition. The Early Dorset (‘Greenlandic Dorset’) expansion into the NOW area (Grønnow and Sørensen 2004) represents a marked human re-settling, not only of the High Arctic, but also all of Greenland. This takes place around 2700 cal. years bp, which means that there is a correspondence with the timing of the expansion of the little auk colony and some other indications of a ‘strong’ NOW polynya. The Dorset groups abandon the High Arctic sometime before 2300 cal. years bp, and this event occurs within the period of rather unstable little auk numbers with likely peaks in numbers reflected at Qeqertaq, but also low numbers suggested at Annikitisoq from 2500 cal. years bp.

Following both these periods of abandonment, in 3800 and 2300 cal. years bp, the palaeodata indicate the bird numbers increased, and by inference, the polynya entered a period of increased size or greater productivity, in 3600 and 2200 cal. years bp, respectively, although the latter may have been closely followed by a decline. There was, however, no return of the human population, indicating the complexity of the relationship between human habitation and environmental conditions.

The following millennia, post-2300 cal. bp is characterised by total human abandonment of the NOW area. According to the little auk data, this period sees low bird abundance. There is some indication of increased bird abundance from around 1500 cal. bp which shows some agreement, at least it precedes the last major demographic events in the NOW area: the re-occupation of NOW by the Late Dorset (c. 1300–700 cal. bp), but there does not appear to any clear correlation with the Thule Culture expansion (Ruin Island Phase, c. 700–500 cal. bp).

## Conclusion

This study is the first to investigate the long-term patterns in the presence, absence, and abundance of seabird colonies in the NOW across multiple locations and to provide direct evidence of the timing of the onset of colony formation of the three of the key sea bird species the region. The data, particularly when synthesised together, provide indirect evidence on the state, or ‘strength’ of the polynya through time. Some remarkable correlations between cold periods, bird arrival, and number, inferred polynya condition, and major human demographic events are evident. We should caution that these are inferred polynya conditions, and that there are no simple one-to-one relations between the polynya and demographic developments. For example, periods where the polynya is inferred to be large and productive appear coincide with the absence of humans. The present study certainly encourages further investigations along the same lines and in collaboration with other disciplines exploring polynya formation.

## Electronic supplementary material

Below is the link to the electronic supplementary material.

Supplementary material 1 (PDF 1,458 kb)

## Footnotes

1 Radiocarbon dates use the proportion of C that is ^14^C to produce a radiocarbon age, with associated errors. However the production of ^14^C depends on solar activity, which has varied over time. Calibrated ages use a range of archives to reconstruct variation in ^14^C over time and therefore allow us to calibrate a ^14^C date to a calendar date. This can be expressed as BC\AD or as age expressed as calibrated years before present or bp.
