# Supplementary material for: The history of seabird colonies and the North Water ecosystem: Contributions from palaeoecological and archaeological evidence
Source: Ambio. 2018 Mar 7;47(Suppl 2):175–92. doi: 10.1007/s13280-018-1031-1 (PMC5963565; doi:10.1007/s13280-018-1031-1)

Ambio

Electronic Supplementary Material

This supplementary material has not been peer reviewed.

Title: **The history of seabird colonies and the North Water ecosystem: Contributions from palaeoecological and archaeological evidence**

Thomas A. Davidson, Sebastian Wetterich, Kasper L. Johansen, Bjarne Grønnow, Torben Windirsc), Erik Jeppesen, Jari Syvyränta, Jesper Olsen, Ivan González-Bergonzoni, Astrid Strunk, Nicolaj K. Larsen, Hanno Meyer, Jens Søndergaard, Rune Dietz, Igor Eulears & Anders Mosbech

Supplementary figures

Figure S1. Depth age model of Bayesian based modelling of ^{14}C dates for NOW5a – Qertertat (Salve Ø)

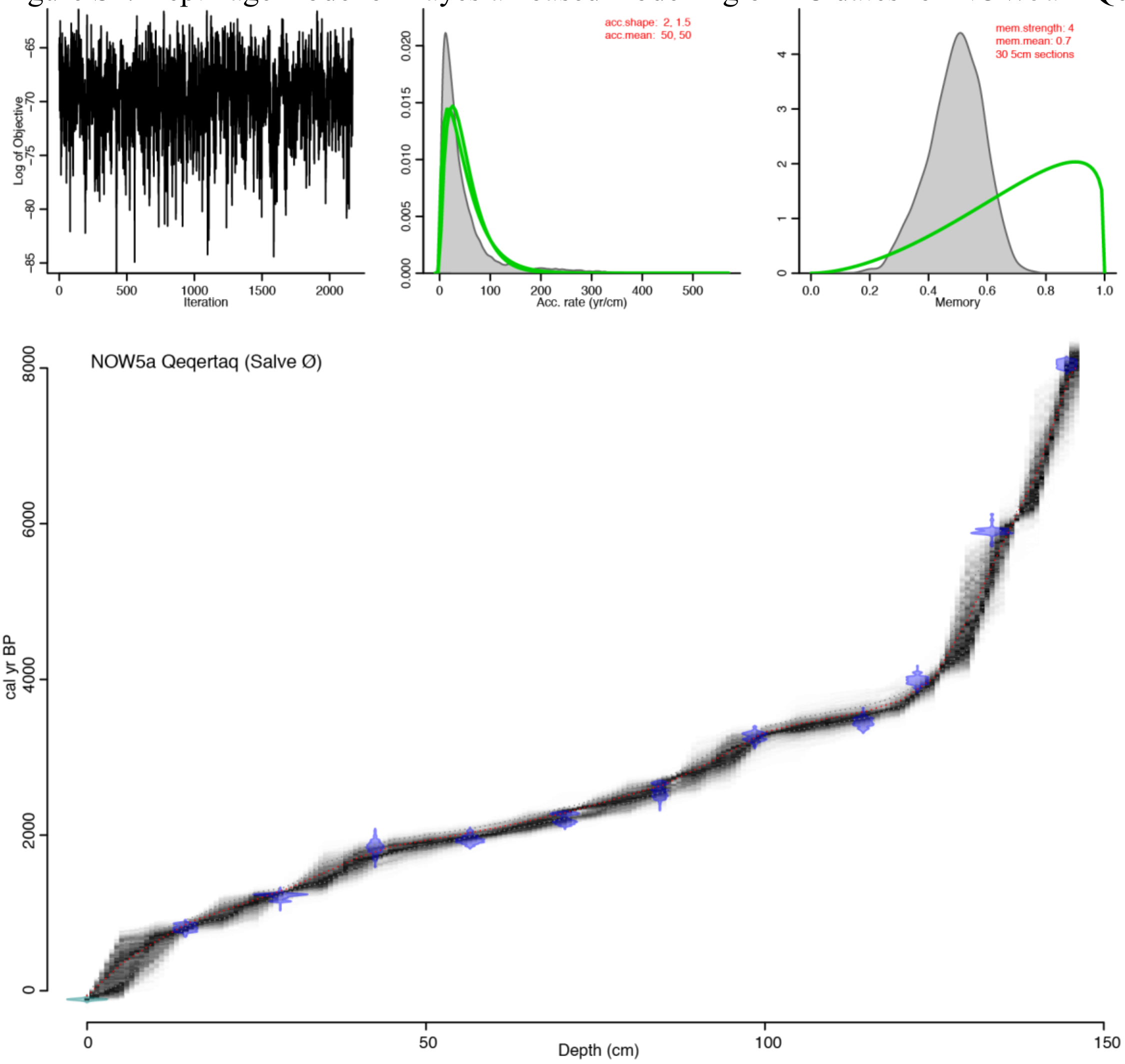

Figure S2. Depth age model of Bayesian based modelling of ^{14}C dates for GL3– Annikitsoq

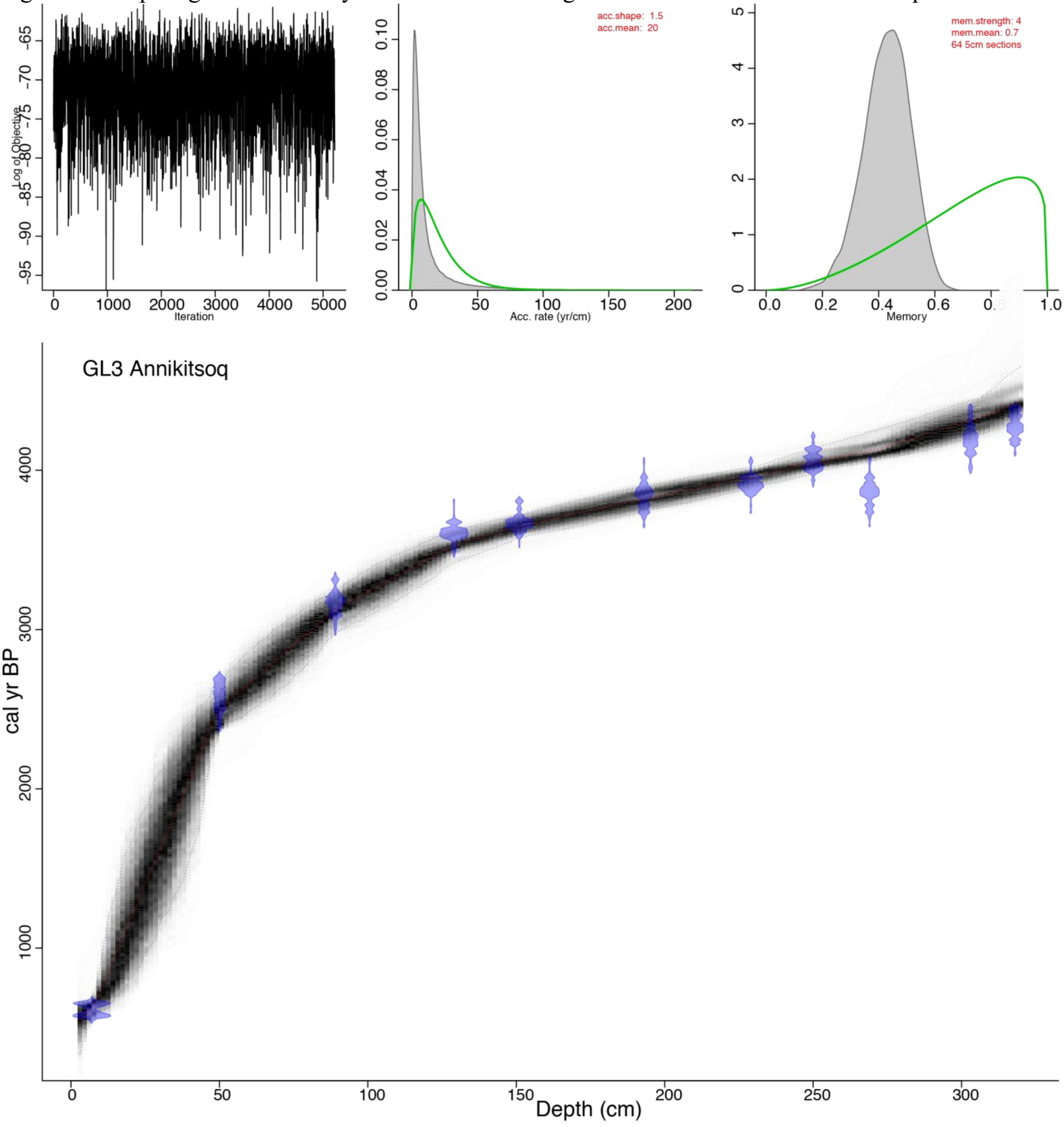

Figure S3. Depth age model of Bayesian based modelling of ^{14}C dates for SD1– Qoororsuaq

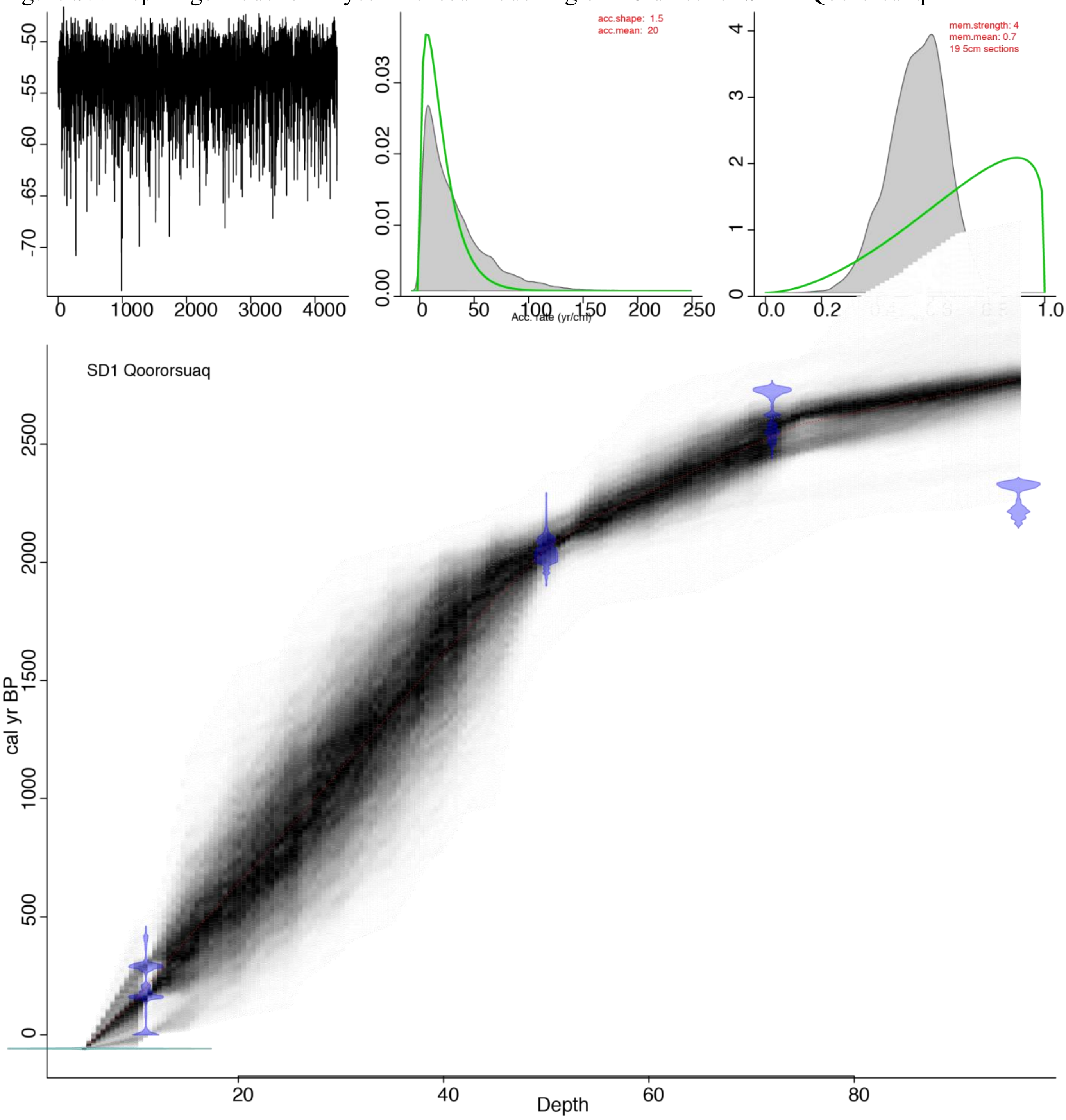

Figure S4. Depth age model of Bayesian based modelling of ^{14}C dates for RF1– Kuukkat

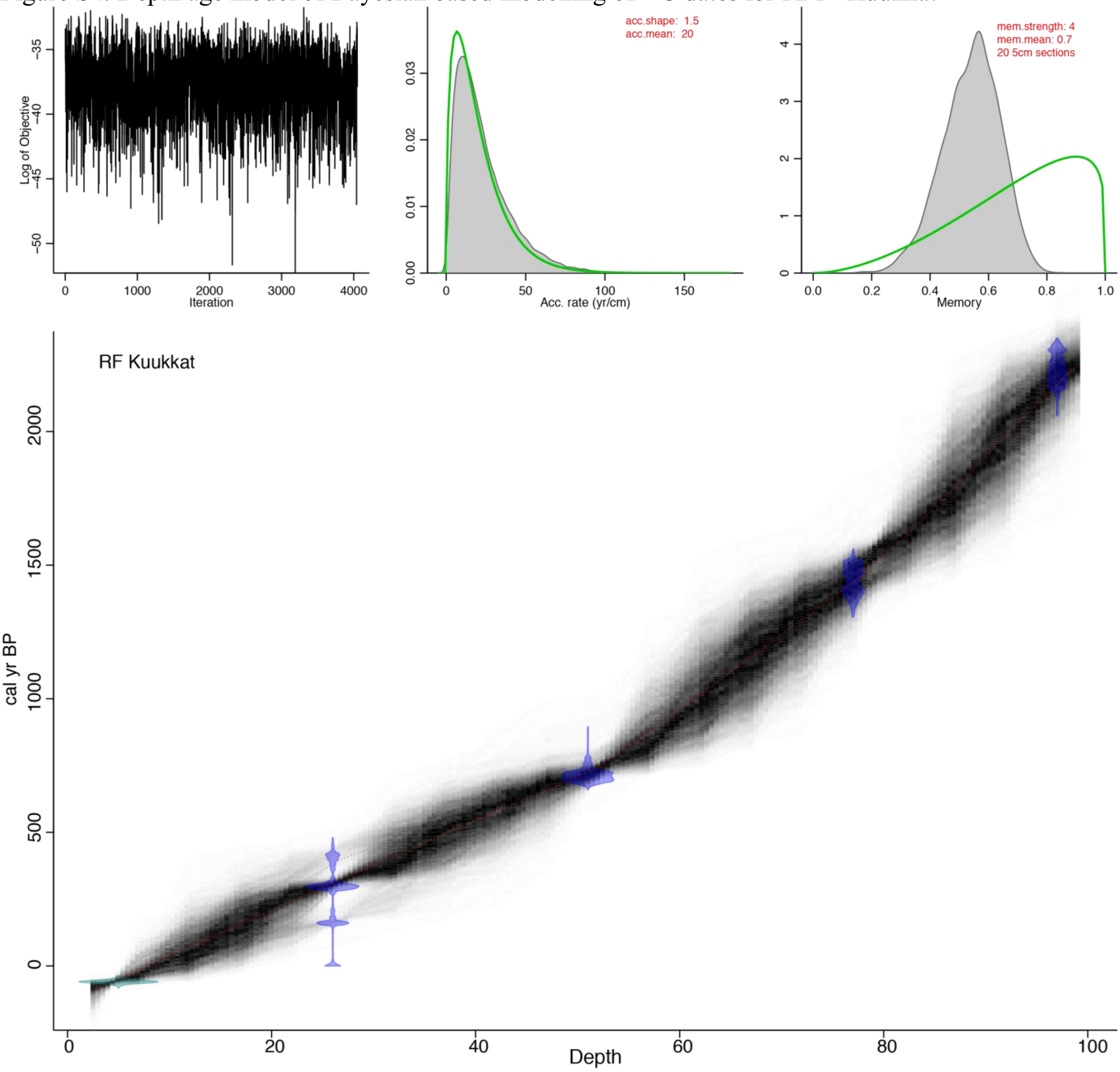

Figure S5. Depth age model of Bayesian based modelling of ^{14}C dates for SI – Appat (Saunders Ø)

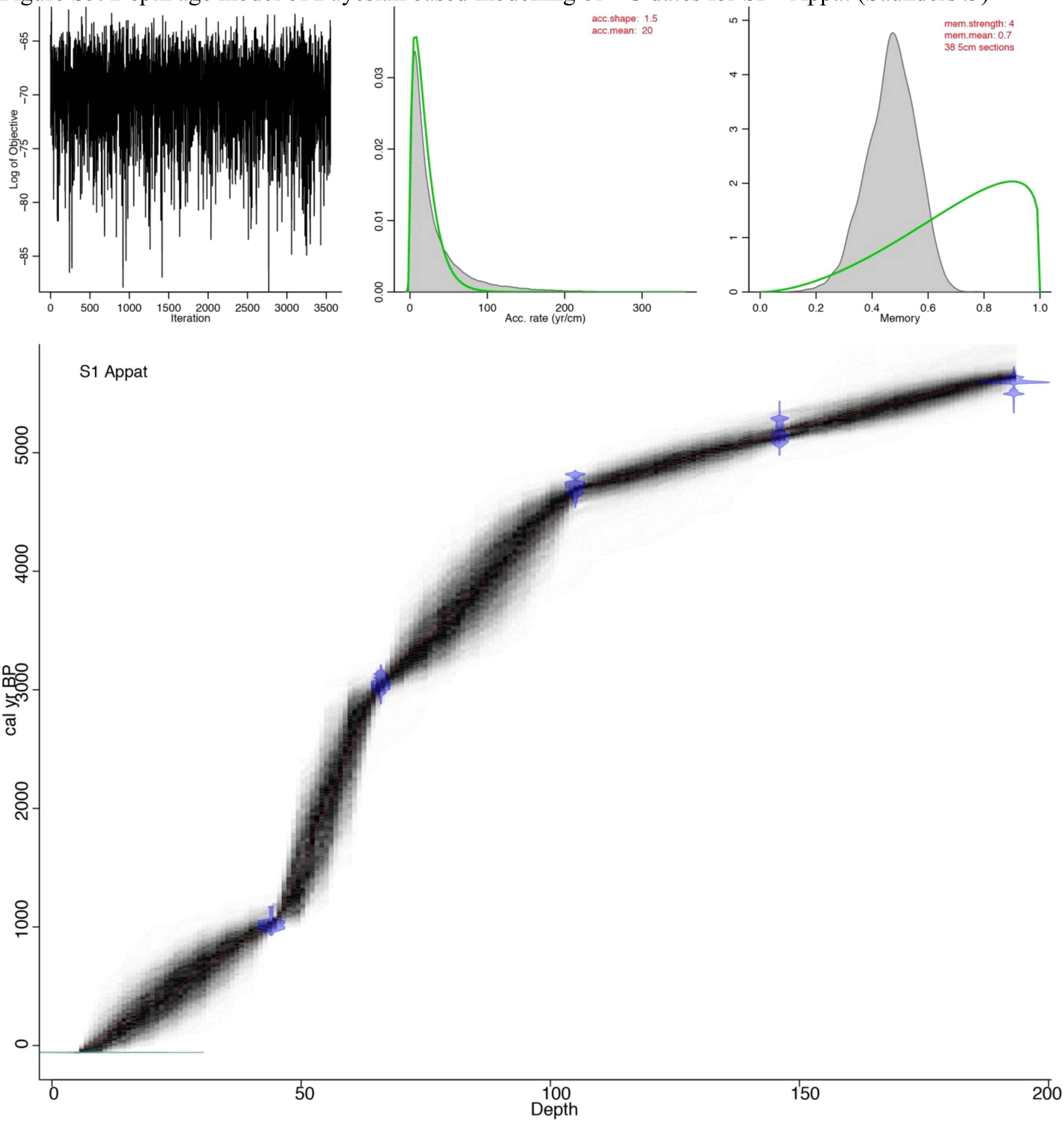

Figure S6. Depth age model of Bayesian based modelling of ^{14}C dates for TSB2 Iterlassuup Qeqertaarsui

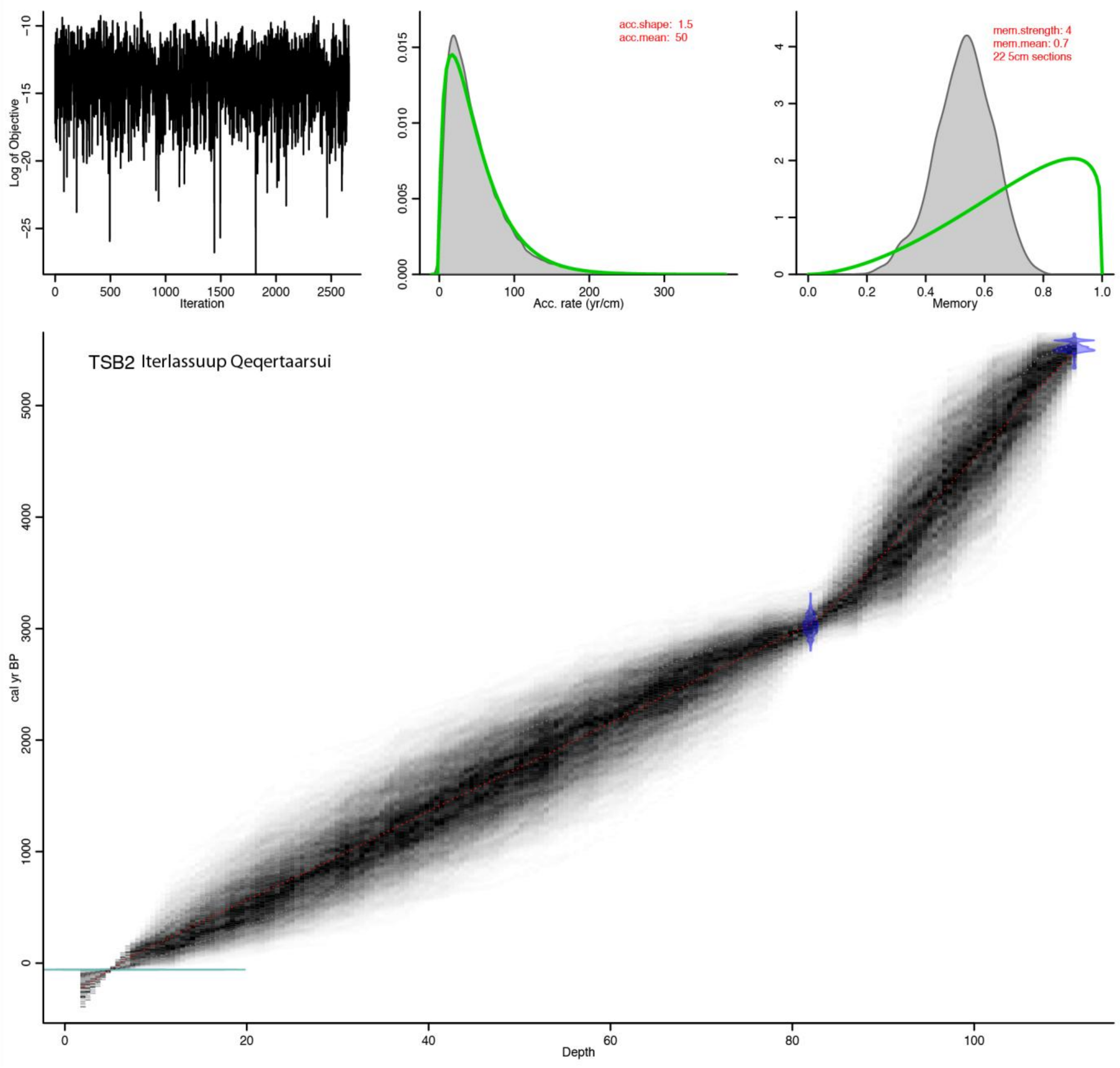

Figure S7. Depth age model of Bayesian based modelling of ^{14}C dates for BS1 Booth Sund

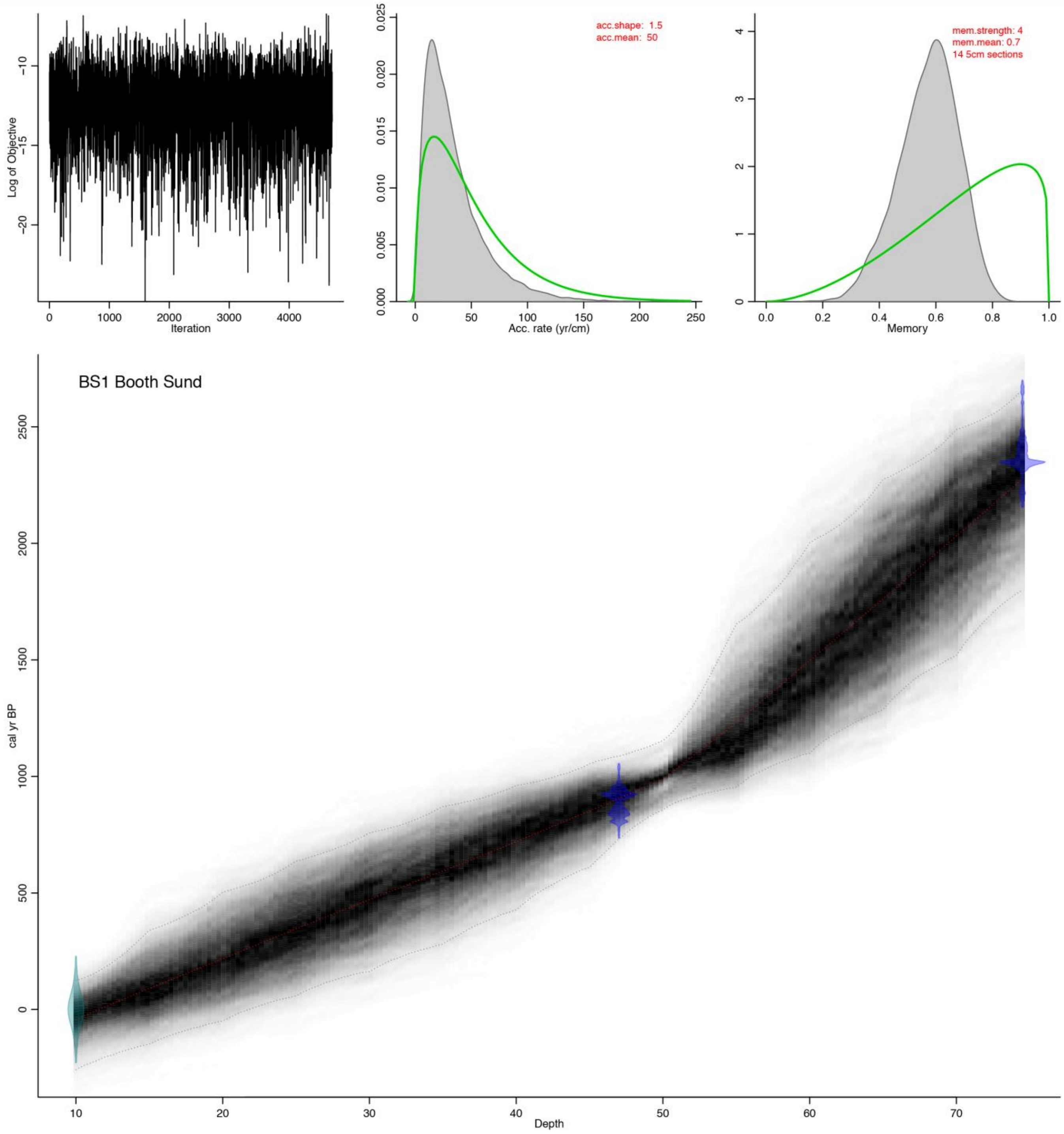

Supplement: Supplementary file 1 — Supplementary material 1 (PDF 1,458 kb) [file 13280_2018_1031_MOESM1_ESM.pdf]
